# Self-isolation of adolescents after Covid-19 pandemic between social withdrawal and Hikikomori risk in Italy

**DOI:** 10.1038/s41598-024-84187-5

**Published:** 2025-01-15

**Authors:** Loredana Cerbara, Giulia Ciancimino, Gianni Corsetti, Antonio Tintori

**Affiliations:** 1 Institute for Research on Population and Social Policies, National Research Council of Italy, 00185 Rome, Italy; 2 Italian National Institute of Statistics, 00184 Rome, Italy

**Keywords:** Psychology, Human behaviour

## Abstract

Social withdrawal is a widespread phenomenon among adolescents, presenting significant challenges in understanding its aetiology and dynamics. This study, drawing on data from two cross-sectional surveys conducted in 2019 and 2022 on students in public upper secondary schools, investigates the trend of self-isolation among Italian adolescents before and after the COVID-19 pandemic. The two nationally representative samples comprise 3273 and 4288 participants, respectively, with 46.3% and 41.2% identifying as female, ranging in age from 14 to 19 years. Using Multiple Correspondence Analysis (MCA) and Hierarchical Cluster Analysis (HCA), three distinct profiles of social withdrawal were identified among adolescents: “Social Butterflies”, “Friendship-Centric”, and “Lone Wolves”. Notably, the discovery of a subgroup within the Lone Wolves, composed of adolescents who never meet their friends and whose number has doubled post-pandemic, highlights the chronic nature of the phenomenon that demands urgent intervention. These findings shed light on the increase of self-isolation in Italy, showing the interplay of socio-demographic, psychological, and sociological factors underlying this phenomenon.

## Introduction

Social withdrawal is a multidimensional phenomenon characterised by voluntary and prolonged self-isolation. It can be considered an umbrella term which refers to the growing manifestation of solitary behaviours, such as spending excessive time alone, avoiding interactions with peers, and experiencing high levels of social anxiety^1^. During adolescence, while temporary and typical experiences of loneliness or a preference for solitude generally do not hinder development, more severe and enduring forms of social withdrawal can significantly impact psychological well-being and developmental trajectories^2^.

Referring to an acute form of social isolation that has spread in Japan since the 1970s, psychiatrist Tamaki Saito coined the term “Hikikomori” defining it as a problem that “involves cooping oneself up in one’s own home and not participating in society for six months or longer, but that does not seem to have another psychological problem as its principal source”^3^.

Initially, severe social withdrawal was believed to be specifically linked to the Japanese context due to the modernization process that characterized the country after World War II and specific sociocultural characteristics unique to that society, such as the close mother-son bond, paternal absence, and the high level of competitiveness in the education system^4,5^. However, several studies conducted in recent decades have reported the spread of this phenomenon in numerous countries worldwide, raising important questions about its nature and the cultural and social influences that may contribute to voluntary social isolation in each context^6–10^.

According to the literature, social withdrawal primarily affects male adolescents and young adults and is associated with an interplay between psychological and social factors, including parenting styles, family dynamics, bullying and cyberbullying victimisation, peer rejection, low self-esteem, and social anxiety^11^. Emerging evidence also highlights the role of body image dissatisfaction as a contributing factor to social withdrawal in adolescence: youth with poor body image are more likely to experience heightened social anxiety, which can lead to avoidance of social interactions and exacerbate feelings of rejection^12^. Furthermore, the tendency towards isolation in advanced capitalist societies is believed to stem from social pressures, as the pursuit of success and competitiveness could lead to social rejection in response to perceived failures^13^.

Considering the pivotal role of peer networks in adolescence, withdrawal from social interactions during this critical stage may hinder the development of social skills and self-esteem, with long-term consequences^14,15^. Adolescence is marked by a heightened need for belonging, as adolescents seek affirmation and acceptance within their peer communities. When these intrinsic developmental needs remain unmet, feelings of isolation may be exacerbated, further reinforcing withdrawal tendencies^16^. In this context, the interplay between online and offline environments emerges as particularly complex. While digital interactions offer adolescents a means to maintain social connections, such interactions often fail to meet the deeper psychological needs for belonging and intimacy, particularly among those with lower social status or histories of peer rejection, thereby exacerbating feelings of exclusion and reinforcing patterns of withdrawal^17^. For this reason, the role of internet use in self-isolation trajectories remains debated, with research alternately framing it as a risk factor or a coping mechanism^18,19^.

The COVID-19 pandemic exacerbated these challenges, with Italy implementing some of the strictest measures globally to mitigate the virus’s spread. Nationwide lockdowns enforced school closures, suspended extracurricular activities, and restricted social gatherings, disrupting adolescents’ social networks and essential peer interactions. Quarantine, on the other hand, imposed strict isolation for individuals exposed to infection, often confining them within households. While lockdown measures led to social distancing from the external world, severing critical peer networks and limiting opportunities for social connection, quarantines forced prolonged cohabitation, sometimes in overcrowded living conditions. This double effect—distancing from peers coupled with enforced proximity to family members—heightened emotional distress, particularly in families with pre-existing conflicts or limited private space^20^. Numerous studies have highlighted that these non-pharmacological strategies, implemented across several countries to protect public health, inadvertently increased risks to adolescents’ mental health, leading to heightened levels of anxiety, depression, and social withdrawal^21–24^.

In Italy, research on social withdrawal primarily focuses on theoretical and qualitative aspects, with an emphasis on psychological factors^25–29^. A recent study aimed at quantifying voluntary isolation among adolescents revealed that approximately 1.7% of Italian students could be classified as Hikikomori, with an additional 2.6% at risk^30^. However, other studies suggest that social withdrawal in Italy manifests in a softer way, involving partial isolation with persistent outside contacts^27,31,32^. The different forms of social withdrawal, as well as its causes, which are strongly tied to cultural contexts^33^, pose challenges in determining its prevalence and undertaking cross-cultural studies^34^. Therefore, further studies are needed to better understand the nuances of the phenomenon across different cultural contexts, taking into account the effects of the pandemic on interactions among adolescents.

With the aim of deepening the understanding of social withdrawal within the Italian context, this study draws on data from two cross-sectional surveys conducted in 2019 and 2022 on representative samples of Italian adolescents to investigate its evolution before and after the COVID-19 pandemic. Furthermore, it profiles individuals based on variables related to leisure time activities, friendship interactions, and concerns about the absence of genuine relationships. To address the multidimensional nature of the phenomenon, we employed an interdisciplinary approach, encompassing a wide range of variables to better understand its dynamics over time. This includes concurrently examining socio-demographic, psychological, and sociological factors linked to social withdrawal.

The following hypotheses have been formulated:

- H1: Physical distancing measures adopted during the pandemic have led to a widespread increase in social withdrawal among Italian adolescents.
- H2: The tendency towards self-isolation can affect any adolescent, regardless of socio-demographic characteristics.
- H3: Adolescents who are at risk of social withdrawal exhibit the following characteristics: poor quality of social relationships, low trust in peers, parental figures, and teachers, high victimisation from bullying and cyberbullying, low self-esteem, low body satisfaction, high hyperconnection, and low involvement in extracurricular activities.

## Results

Table 1 compares the frequency distribution of variables used for multiple correspondence analysis in 2019 and 2022, with detailed descriptions provided in the measures section. In both samples, respondents predominantly spend their leisure time with friends. However, a notable decline in this habit is recorded between 2019 and 2022 (− 14%), along with a slight decrease in time spent with parents (− 6%). Conversely, an increase is observed in the proportion of respondents spending leisure time with friends online (+ 9.7%) and alone (+ 5.7%).

Regarding the frequency of hanging out with friends, meeting friends twice or more a week remained the most prevalent choice in both samples, with a decrease of 7.7% between 2019 and 2022. On the other hand, the proportion of those meeting their friends once a week increased (+ 4.1%), along with an increase in those who never meet them (+ 4.1%). Finally, respondents’ concern about the absence of sincere relationships remained relatively stable between 2019 and 2022 (− 0.9%).

**Table 1:** Frequency of variables used for multiple correspondence analysis (years 2019 and 2022).

		2019		2022	
		*n*	%	*n*	%
Leisure time (people)	Parents (TL_P)	1335	40.8	1491	34.8
	Siblings (TL_S)	963	29.4	1346	31.4
	Partner (TL_PT)	750	22.9	794	18.5
	Friends (online) (TL_FO)	929	28.4	1635	38.1
	Friends (in person) (TL_FP)	2605	79.6	2815	65.6
	Alone (TL_A)	531	16.2	941	21.9
Frequency of hanging out with friends	Never (FR_N)	184	5.6	414	9.7
	Once a week (FR_O)	829	25.3	1250	29.2
	Twice or more a week (FR_T)	1777	54.3	2000	46.6
	Everyday (FR_E)	483	14.8	624	14.6
Main youth issue	Lack of sincere relationships (SR)	394	12.0	476	11.1

Table 2 presents the distribution of respondents who spend their leisure time alone, never meet their friends, and express concern about the absence of sincere relationships across various socio-demographic variables: sex, geographical area, citizenship, and type of school.

The percentages of those spending leisure time alone and those who never meet their friends have increased for both males and females, with females showing a higher proportion in 2022. Concern about the lack of sincere relationships has remained relatively stable and is higher among females. Observing the geographical distribution of these variables, the southern and northeastern regions exhibit the most significant increases in leisure time spent alone and absence of meetings with friends. Italian adolescents generally show lower percentages in these categories compared to their foreign counterparts, although both groups have experienced increases from 2019 to 2022. Moreover, Lyceum students consistently show higher percentages in spending leisure time alone and concern about the absence of sincere relationships, whereas technical and vocational school students tend to report higher rates of lack of weekly meetings with friends.

**Table 2:** Frequencies of variables used for multiple correspondence analysis across socio-demographic variables (years 2019 and 2022).

	Leisure time alone		Lack of meetings with friends		Lack of sincere relationships	
	2019 (%)	2022 (%)	2019 (%)	2022 (%)	2019 (%)	2022 (%)
Sex						
Male	16.7	21.2	5.0	8.8	10.7	10.5
Female	16.4	23.0	6.3	10.9	13.6	12.1
Geographical area						
South	9.9	19.0	5.7	10.3	11.7	11.1
Centre	15.0	17.5	5.6	7.5	13.2	12.9
North East	21.7	30.6	5.2	11.0	11.1	10.1
North West	18.6	23.4	6.0	9.1	12.1	10.4
Citizenship						
Italian	15.9	21.5	5.1	8.9	12.5	11.2
Foreign	22.9	27.2	10.4	19.8	7.7	10.6
Type of school						
Lyceum	20.6	28.5	3.8	7.1	13.7	12.8
Technical institute	16.2	18.5	6.0	10.1	11.6	10.8
Vocational school	11.8	16.9	7.4	12.6	10.5	9.3

### MCA results

In this section, we describe the 4 dimensions, 2 for each year, obtained from applying the Multiple Correspondence Analysis (MCA) technique to both datasets. Tables 3 and 4 delineates the coordinates of variables employed in the MCA, along with their contributions which facilitate the identification of the most significant variables influencing the axes, as they better elucidate variations across data.

The first dimension in 2019, primarily pertains to social interactions with friends and tendencies towards isolation during leisure time, explaining 58.3% of the variance. Positive contributions indicate a preference for spending leisure time in solitary activities and low weekly frequencies of meetings with friends (i.e., never or once a week). Conversely, negative values on this dimension suggest a tendency to spend leisure time with friends in person and maintain a high weekly frequency of meetings with them (i.e., every day or at least twice a week). The second dimension selected for 2019 explains the 40.1% of the variance and mainly pertains to interactions with family members. Positive contributions arise from the tendency not to spend leisure time with parents and siblings, while negative contributions indicate the habit of spending leisure time with them.

Unlike the clear distinction between friends and family observed in 2019, the selected dimensions for 2022 reveal a more intertwined landscape. The first dimension for 2022, explaining 67.4% of the variance, can be interpreted as a comprehensive propension towards isolation, encompassing both family and friends. Significant positive contributions include avoiding spending leisure time with friends, parents and siblings, lack of weekly meetings with friends, and a preference for solitary activities. Conversely, noteworthy negative contributions stem from spending leisure time with family and friends in person. Lastly, the second dimension in 2022, explains 29.6% of the variance and relates to the inclination to spend leisure time either solely with family, relegating interactions with friends to virtual platforms, or solely with friends. Major positive contributions entail spending leisure time with parents and siblings, as well as engaging with friends exclusively online, along with solitary activities and a low frequency in meetings with friends (i.e., never or once a week). The main negative contribution, on the other hand, arises from daily meetings with friends.

**Table 3:** MCA results (year 2019).

	2019			
	Dim1		Dim2	
	Coordinates	Contributions	Coordinates	Contributions
Leisure time (people)				
Parents (no)	− 0.184	1.244	0.623	15.251
Parents (yes)	0.267	1.805	− 0.905	22.140
Siblings (no)	− 0.147	0.939	0.461	9.941
Siblings (yes)	0.351	2.253	− 1.106	23.846
Partner (no)	0.109	0.571	− 0.032	0.053
Partner (yes)	− 0.368	1.921	0.108	0.178
Friends (online) (no)	− 0.216	2.069	0.174	1.433
Friends (online) (yes)	0.545	5.220	− 0.438	3.615
Friends (in person) (no)	1.274	20.530	0.648	5.686
Friends (in person) (yes)	− 0.327	5.265	− 0.166	1.458
Alone (no)	− 0.260	3.512	− 0.097	0.527
Alone (yes)	1.343	18.134	0.503	2.722
Frequency of hanging out with friends				
Never	2.095	15.289	1.341	6.701
Once a week	0.791	9.823	− 0.136	0.310
Twice or more a week	− 0.342	3.934	− 0.248	2.208
Everyday	− 0.898	7.372	0.634	3.928
Main youth issue				
Lack of sincere relationships (no)	− 0.016	0.014	0.002	0.000
Lack of sincere relationships (yes)	0.119	0.105	− 0.017	0.002

**Table 4:** MCA results (year 2022).

	2022			
	Dim1		Dim2	
	Coordinates	Contributions	Coordinates	Contributions
Leisure time (people)				
Parents (no)	0.453	7.868	− 0.362	5.846
Parents (yes)	− 0.849	14.760	0.679	10.967
Siblings (no)	0.400	6.465	− 0.344	5.547
Siblings (yes)	− 0.874	14.131	0.752	12.124
Partner (no)	0.055	0.143	0.157	1.371
Partner (yes)	− 0.240	0.629	− 0.691	6.035
Friends (online) (no)	0.147	0.789	− 0.334	4.722
Friends (online) (yes)	− 0.239	1.280	0.542	7.663
Friends (in person) (no)	0.910	16.767	0.487	5.570
Friends (in person) (yes)	− 0.476	8.773	− 0.255	2.915
Alone (no)	− 0.207	1.965	− 0.197	2.079
Alone (yes)	0.735	6.989	0.702	7.395
Frequency of hanging out with friends				
Never	1.481	12.467	0.952	5.984
Once a week	0.302	1.564	0.602	7.209
Twice or more a week	− 0.411	4.635	− 0.218	1.516
Everyday	− 0.270	0.627	− 1.138	12.875
Main youth issue				
Lack of sincere relationships (no)	0.018	0.016	− 0.018	0.020
Lack of sincere relationships (yes)	− 0.142	0.132	0.145	0.160

## Results of hierarchical cluster analysis

Through Hierarchical Cluster Analysis, three homogeneous groups of adolescents were identified for each year: Social Butterflies, comprising 35.2% of the sample in 2019 and 38.5% in 2022; Friendship-Centric, which decreased from 49% of the sample in 2019 to 22.1% in 2022; and Lone Wolves, which included 15.8% of the sample in 2019 and 39.4% in 2022. Table 5 illustrates the clusters’ composition according to various socio-demographic variables. The Social Butterflies cluster is composed of adolescents who exhibit high levels of social interaction with both friends and family members, spending their leisure time with them. However, while in 2019 the members of this group tended to meet their friends in person and very frequently during the week, in 2022 the frequency of meetings with friends has reduced, and interaction with them is mostly relegated to the virtual sphere. In 2019, this group showed a higher proportion of females, lyceum students, with no difference between Italians and foreigners. A smaller proportion was recorded among students from the northeast and those who do not engage in sports. In 2022, this cluster is somewhat different in its composition: in this case, there is a higher proportion of males and Italian students, while the lower presence of students from the northeast as well as the higher proportion of lyceums student persist.

The Friendship-Centric cluster is characterised by adolescents who prioritise social interactions with their peers, particularly in-person meetings with friends, while not spending their leisure time with parents or siblings. It was the largest group in 2019 and the smallest in 2022. In 2019, this cluster showed a relatively higher proportion of males, students with Italian citizenship, and students attending vocational schools. In this case, the proportion of students from the northeast is higher compared to other macro areas. In 2022, similarly to the previous group, the composition of this group changes, and we observe a higher proportion of females, adolescents from the central regions, and a wider disparity in terms of citizenship, with a higher presence of Italian students.

Finally, the Lone Wolves cluster is characterised by individuals who primarily spend their leisure time alone and, therefore, neither with their family nor peers, showing a low weekly frequency in in-person meetings with friends. The composition remains almost the same in both years: a higher proportion of females, students from northern regions of Italy, and foreign citizens. Regarding the type of school, the proportion of lyceum students is the highest for both years, but in 2022, it is accompanied by that of students from vocational schools. Within this group are included all students who never meet their friends, who amounted to 5.6% of the sample in 2019 and 9.7% in 2022, as illustrated at the beginning of the results description.

**Table 5:** Clusters’ composition across socio-demographic variables (years 2019 and 2022).

	Social butterflies		Friendship-centric		Lone wolves	
	2019 (35.2%) n 1152 (%)	2022 (38.5%) n 1651 (%)	2019 (49.0%) n 1605 (%)	2022 (22.1%) n 948 (%)	2019 (15.8%) n 516 (%)	2022 (39.4%) n 1689 (%)
Sex						
Male	33.5	39.7	53.2	24.2	13.3	36.1
Female	37.1	36.8	44.3	19.1	18.6	44.1
Geographical area						
South	36.4	37.3	49.7	23.3	14.0	39.4
Centre	37.2	40.5	47.7	24.5	15.1	35.0
North East	32.2	36.5	51.0	19.2	16.8	44.3
North West	35.3	40.9	47.8	20.3	16.9	38.7
Citizenship						
Italian	35.2	38.9	49.7	22.8	15.1	38.3
Foreign	34.8	33.9	42.8	12.8	22.4	53.4
Type of school						
Lyceum	38.8	41.3	43.8	17.3	17.4	41.4
Technical institute	35.0	40.0	50.5	24.2	14.5	35.8
Vocational school	31.1	33.1	53.9	26.2	15.1	40.7

Table 6 illustrates the distribution of psychological and sociological variables across the three clusters of adolescents. Social Butterflies consistently show lower proportions of poor relationship quality with both fathers and mothers and higher levels of relational trust compared to the other clusters, both in 2019 and 2022. In terms of individual well-being, while the percentage of adolescents with low self-esteem is the lowest among Social Butterflies in 2019, it significantly increases by 2022, exceeding that of the Friendship-Centric cluster. Moreover, within this cluster, the proportion of hyperconnection doubles, becoming the highest in 2022. Additionally, there is a notable uptick in cyberbullying instances and dissatisfaction with body image.

The Friendship-Centric cluster exhibits a lower quality of relationships with parents and lower relational trust compared to Social Butterflies, with proportions similar to those of the Lone Wolves. Particularly noteworthy is the low level of trust in teachers within this cluster, with approximately six out of ten members expressing little or no trust in them. Levels of self-esteem remain relatively stable between 2019 and 2022, while the percentage of (cyber)bullying victimisation is the lowest among the three clusters. Similar to Social Butterflies, there is a significant increase in time spent online, coupled with a decline in participation in sports and body satisfaction.

Lone Wolves consistently show the highest proportions of poor relationship quality with parents and low relational trust compared to other clusters. They also exhibit the highest prevalence of low self-esteem, dissatisfaction with body image, and (cyber)bullying victimisation. However, despite the increase in the proportion of hyperconnected members over time, they exhibit the lowest rate of time spent on social media compared to the other two clusters.

**Table 6:** Proportions of psychological and sociological variables within clusters (years 2019 and 2022).

		Social butterflies (%)	Friendship-centric (%)	Lone wolves (%)	*N*
Poor relationship quality					
Father	2019	12.9	21.8	23.0	18.8% (n 602)
	2022	15.7	23.4	25.1	21.1% (n 880)
Mother	2019	10.1	17.7	17.4	14.9% (n 487)
	2022	14.4	18.1	20.7	17.7% (n 756)
Low relational trust					
Father	2019	10.0	16.0	19.7	14.4% (n 470)
	2022	12.5	16.4	22.6	17.3% (n 724)
Mother	2019	2.4	9.0	8.9	6.7% (n 217)
	2022	6.6	9.9	15.0	10.6% (n 453)
Other family members	2019	8.5	17.5	21.8	14.9% (n 487)
	2022	14.8	21.9	28.5	21.8% (n 935)
Friends	2019	7.7	11.1	29.1	12.7% (n 416)
	2022	14.2	15.3	29.1	20.3% (n 872)
School teachers	2019	46.8	56.0	47.6	51.4% (n 1677)
	2022	53.7	62.6	57.3	57.1% (n 2448)
Individual well being					
Low self-esteem	2019	18.6	25.3	36.2	24.6% (n 807)
	2022	28.3	27.6	43.3	34.1% (n 1461)
Time on social media					
Hyperconnection	2019	19.9	25.2	23.8	23.1% (n 756)
	2022	40.7	40.1	37.8	39.4% (n 1691)
Sport practice					
Yes	2019	69.4	68.2	52.9	66.2% (n 2168)
	2022	67.2	63.7	52.2	60.5% (n 2595)
(Cyber)bullying victims					
Yes	2019	49.9	47.0	63.0	50.6% (n 1655)
	2022	58.8	51.4	66.9	60.3% (n 2587)
Body satisfaction					
Yes	2019	66.7	65.2	54.5	64% (n 2095)
	2022	58.6	60.3	50.7	55.9% (n 2396)

Table 7 compares proportions of specific social and individual variables between 2019 and 2022 within the Lone Wolves cluster, showing the significance of the differences between the two samples. The results reveal important variations concerning both the relational and individual well-being of adolescent, as well as some social phenomena, such as hyperconnection and (cyber)bullying. Regarding the poor relationship quality with parents, differences in proportion between 2019 and 2022 suggest a worsening trend, especially in mother-son relationships. Furthermore, among group members, there is a significant decrease in trust towards mothers, other family members, and teachers. The proportion of adolescents with low self-esteem within the cluster increases, indicating a deterioration of individual well-being. Lastly, there is a significant increase in the proportion of hyperconnected adolescents and victims of bullying and cyberbullying.

**Table 7:** Comparison of proportions and variations over time in lone wolves cluster.

		Proportions	Difference in proportions	Asymptotic standard error	*p*-value
Poor relationship quality					
Father	2019	0.23	− 0.02	0.02	0.17
	2022	0.25			
Mother	2019	0.17	− 0.03	0.02	0.05
	2022	0.21			
Low relational trust					
Father	2019	0.20	− 0.03	0.02	0.08
	2022	0.23			
Mother	2019	0.09	− 0.06	0.02	0.00
	2022	0.15			
Other family members	2019	0.22	− 0.07	0.02	0.00
	2022	0.29			
Friends	2019	0.29	0.00	0.02	0.50
	2022	0.29			
School teachers	2019	0.48	− 0.10	0.03	0.00
	2022	0.57			
Individual well being					
Low self-esteem	2019	0.36	− 0.07	0.02	0.00
	2022	0.43			
	2022	0.13			
Time on social media					
Hyperconnection	2019	0.24	− 0.14	0.02	0.00
	2022	0.38			
Sport practice					
Yes	2019	0.53	0.01	0.03	0.38
	2022	0.52			
(Cyber)bullying victims					
Yes	2019	0.63	− 0.04	0.02	0.05
	2022	0.67			
Body satisfaction					
Yes	2019	0.46	− 0.04	0.03	0.07
	2022	0.49			

## Discussion and conclusion

As numerous international studies have found, the COVID-19 pandemic has significantly impacted human behaviour and social interactions^35–37^. The notable decrease in the number of adolescents spending leisure time with friends between 2019 and 2022 is not incidental. Indeed, this trend towards self-isolation is probably one of the consequences of physical distancing measures and lockdowns adopted to contain the virus’ spread. The increase of hyperconnection was pushing adolescents towards the transposition of human relationships towards the virtual sphere already before the pandemic. However, in the absence of this disruptive event, such a sudden and significant transformation would not have even been predictable. Thus, the spread of COVID-19 has accelerated this phenomenon, exacerbating the trend towards self-isolation. As observed, from 2019 to 2022, the prevalence of Italian adolescents spending more than three hours a day on social media has doubled, while the habit of spending leisure time with friends, as well as the weekly frequency of face-to-face meetings with friends have significantly decreased. The study’s findings confirm the validity of our first hypothesis (H1), showing a chronicity of self-isolation as a psychosocial effect of the COVID-19 spread.

The HCA’s results fully support also the second hypothesis (H2). Even though the phenomenon is slightly more prevalent among girls, it encompasses both genders, adolescents across different Italian regions, and students from all types of schools who have different socio-cultural and economic backgrounds. Therefore, the problem is increasingly worrying because it is becoming global and endemic. As we have seen, the cluster analysis delineated three distinct social groups with markedly different relational patterns. However, the clusters’ diachronic comparison shows an overall deterioration of relationships’ quality and a decrease in relational trust from 2019 to 2022. This decline also affects the Social Butterflies group, whose members are the most sociable among the respondents. Beyond these general trends, the greatest concern arises from the Lone Wolves. The remarkable increase of adolescents belonging to this group - tripling in 3 years from 15 to 39.4% - is the most pronounced indication of the widespread nature of the phenomenon. The Lone Wolves group is a discovery of great significance because its examination has shed light on the main factors associated to social isolation: poor relationship quality, low relational trust, and victimisation from cyberbullying and bullying. The composition of this subgroup has remained consistent across both years of analysis, with a higher representation of females, students from Northern Italy, and foreign citizens. Regarding school types, while lyceum students form the largest share of this group, the 2022 data reveal a notable increase in the proportion of students from vocational schools, suggesting a potential broadening of the phenomenon across diverse educational contexts. These findings suggest the need for a deeper exploration of how demographic, cultural, and individual factors intersect to influence the experience of social withdrawal. through further longitudinal studies conducted in various contexts. Monitoring these trends and defining the causal factors of youth social withdrawal with increasing accuracy will be essential. Delving into the dynamics of the gradual disaffection from direct human relationships that lead adolescents towards total abandonment of social interactions with the outside world is crucial. This condition, exacerbating the phenomenon, aligns with the concept of Hikikomori.

Moreover, the findings regarding the role of hyperconnection challenge some aspects of the third hypothesis (H3). Specifically, there has been a general increase in time spent on social media usage, doubling between 2019 and 2022, which has been observed among both Social Butterflies and Friendship-Centric members. Contrary to our expectations based on existing literature, a similar increase in social media use was not observed among the Lone Wolves, who reported even lower levels of social media engagement in 2022. This unexpected finding could be a reaction to previous negative experiences both offline and online, which generate fear towards peer interaction, thereby reducing access to social media. It is also possible that as the duration of physical isolation increases and face-to-face interactions become more detached, adolescents gradually disconnect from social media over time, withdrawing from virtual environments as well.

Unfortunately, the available survey variables do not allow us to verify this hypothesis, as data about time spent on social media prior to the interviews were not collected. However, this consideration may become the basis for a new hypothesis to be tested using statistically representative data.

Finally, in relation to the third hypothesis (H3), two other factors are associated with the trend towards social withdrawal: low participation in extracurricular sports practice and dissatisfaction with one’s body. Sport is known to be a significant factor in expanding peer networks, also contributing to the quality of friendships^38^. Conversely, dissatisfaction with body image reflects the pervasive influence of societal pressures to conform to unattainable aesthetic standards, disproportionately affecting girls and contributing to a greater propensity for self-isolation. Negative body perceptions often exacerbate vulnerability to social withdrawal by eroding self-confidence and fostering a sense of inadequacy in social interactions with peers. This study has some limitations that warrant consideration. Social withdrawal is an emerging phenomenon that has only recently begun to receive more in-depth academic attention. To gain a more comprehensive understanding of this issue, future research should incorporate specific variables that were absent in our study, such as the history of past episodes of isolation, their duration and intensity, the activities conducted during these periods, and the nature of family relationships during such times. Furthermore, the cross-sectional design of this study limits the ability to establish robust causal relationships between the identified factors and social withdrawal. Longitudinal studies are therefore essential to explore the dynamics of this phenomenon over time and to provide a clearer picture of its development and influencing factors. In conclusion, to gain a better understanding of social withdrawal it is crucial to delve into both its aetiology and its developmental processes that increasingly lead young people to isolation over time. Urgent intervention is necessary at both the school and family levels, as well as through personal professional support, for the subgroup of adolescents currently in the most critical conditions. This subgroup, part of the Lone Wolves cluster, consists of students who no longer meet their friends outside of school. As shown in the study results, the numbers of this subgroup have doubled from 2019 to 2022 (from 5.6 to 9.7%), raising concerns that this group, once they complete compulsory schooling, may increasingly resemble Japan’s Hikikomori phenomenon. They exhibit poor-quality relationships with parents, particularly with the mother, low trust towards family members and teachers, and low self-esteem.

Future studies will allow us to understand if adolescents with the characteristics of this subgroup will eventually be assimilated to the Japanese phenomenon of hikikomori. However, if the number of these young people continues to grow, Western countries with advanced economies will face an unprecedented social emergency. By enriching the understanding of the Lone Wolves subgroup, this study opens avenues for more nuanced research and evidence-based strategies to mitigate the alarming rise of youth social withdrawal. Future research should prioritize longitudinal designs and cross-context analyses to validate these findings and explore the intersection of cultural, institutional, and personal dynamics contributing to this complex phenomenon. From a practical perspective, understanding the specific needs of the Lone Wolves subgroup is paramount for designing targeted interventions. The data suggest that this group requires support strategies addressing the deterioration of relational trust and the impacts of bullying, both online and offline. Moreover, their gradual disconnection from direct human relationships—a trajectory reminiscent of the Hikikomori phenomenon—underscores the need for early and comprehensive interventions to prevent complete social withdrawal. Such interventions should include efforts to rebuild trust, foster positive peer relationships, education in the use of the virtual world and social media, and provide accessible resources for emotional and psychological support within both school and community settings.

## Materials and methods

### Sample

The current study is based on data from two national surveys conducted in Italy among adolescents enrolled in public upper secondary schools, which in the Italian educational system typically includes students aged 14 to 19 years and span a duration of five years. The study received approval from the Research Ethics and Integrity Committee of the Italian National Research Council on [approval date: 22 July 2021]. In both surveys, a two-stage stratified sampling approach was employed to ensure representative samples at the national level. For the 2019 survey, the Italian territory was initially divided into four main geographical macro-areas: North-West, North-East, Centre, and South. In the 2022 survey, the sampling was expanded, and the territory was divided into five geographical areas: North-West, North-East, Centre, South, and Islands. In both surveys, the first stage involved the random selection of three large cities (with at least 100,000 inhabitants) from each geographical area, resulting in 12 cities in 2019 and 15 cities in 2022. In the second stage, schools were randomly selected from the official lists provided by the Italian Ministry of University and Research (MUR), resulting in a total of 36 schools in 2019 and 45 in 2022. Within each school, five classes—one for each grade—were randomly chosen, for a total of 180 classes in 2019 and 225 in 2022. The final representative samples comprised 3,273 and 4,288 respondents, with 46.3% and 41.2% being female. Table 8 provides additional demographic information about the participants.

**Table 8:** Samples composition across socio-demographic variables.

	2019	2022
	*N* = 3273 (%)	*N* = 4288 (%)
Sex		
Male	53.7	58.8
Female	46.3	41.2
Geographical area		
South	22.9	39.5
Centre	25.6	20.0
North East	26.7	20.1
North West	24.7	20.4
Class		
I	20.8	21.9
II	19.8	20.6
III	21.4	19.2
IV	19.7	19.6
V	18.3	18.7
Citizenship		
Italian	90.9	92.7
Foreign	9.1	7.3
Type of school		
Lyceum	37.2	38.9
Technical institute	32.4	31.9
Vocational school	30.4	29.2
Parental cultural status		
Low	18.1	19.6
Medium	53.8	49.2
High	28.0	31.2

### Procedures

Both surveys were conducted through the administration of a semi-structured electronic questionnaire using the Computer Assisted Personal Interviewing (CAPI) method. Researchers were present in each classroom during the questionnaire administration, which lasted approximately one hour. Specifically, a unique access link to the questionnaire was provided to each class, and students were able to seek clarifications or express any doubts at any point during the process. The presence of research team members within classrooms during the administration process ensured high reliability of the collected data by facilitating respondents’ understanding of the questions, resulting in no missing data and mitigating potential bias from teacher influence. Informed consent was obtained from all participants and their parents or legal guardians, and the voluntary and anonymous nature of participation was emphasised. The electronic questionnaires consisted of 57 and 77 questions respectively, covering various dimensions of analysis, including socio-economic family background, demographic information, family and friendship relationships, lifestyle, leisure activities, offline and online interactions, social media use, deviant behaviours, beliefs, values, relational and systemic trust, and individual well-being. The study received approval from the Research Ethics and Integrity Committee of the Italian National Research Council on [approval date: 22 July 2021]. All methods of the present study were carried out in accordance with relevant guidelines and regulations.

### Measures

#### Frequency of hanging out with friends

The frequency of in-person peer interactions was measured using a 4-point Likert scale, where respondents indicated how often they met their friends during a typical week. Response options ranged from “never” to “every day”.

#### Leisure time

Respondents were asked to indicate with whom they primarily spend their leisure time through a multiple-choice question. The response options included: Parents, Siblings, Partner, Friends (online), Friends (in person), and Alone.

#### Concern about lack of authentic relationships

In order to detect adolescents’ perceptions of the key challenges faced by youth, respondents selected the main issue affecting young people today from a pre-defined list, including: Employment search, State inefficiency, Addictions (drugs, alcohol, gambling), Bullying and cyberbullying, Sexuality, Lack of authentic social relationships, Relationships with parents, Depression and distress, Eating disorders, and Romantic relationships. Through recoding, a dummy variable was constructed to identify respondents who consider the lack of authentic social relationships as the primary issue among youth.

#### Friendship network size

The size of participants’ friendship networks was assessed by asking respondents to report the number of their close friends. Respondents selected one of four predefined categories: zero, one to three, four to six, or more than six close friends. These categories were chosen to provide a manageable and interpretable measure of social connectedness, while capturing meaningful variation in network size. This variable offers a straightforward and reliable indicator of perceived social connections. Parent-child relationship quality. Two variables were employed to assess the quality of relationships between respondents and their parents. One variable focused on the relationship with fathers, while the other on the relationship with mothers, by employing the semantic differential technique. These variables included four pairs of adjectives with opposing meanings to describe the parent-child relationship: Cold/Affectionate, Conflictual/Peaceful, Authoritarian/Permissive, Morbid/Balanced. Each response was recoded on a scale from − 3, indicating proximity to the negative adjective, to 3, representing proximity to the positive adjective, with zero signifying neutrality. The mean score for each relationship was calculated and used to classify the quality of the parent-child relationship into three categories: scores of 0 or below indicated “poor” relationship quality, scores between 0.1 and 2 were classified as “medium,” and scores between 2 and 3 represented “high” relationship quality. Relational trust. Relational trust was assessed by asking respondents how much trust they placed in the following figures at the time of the interview: mother, father, other family members, friends, and teachers. Responses were recorded on a 4-point scale ranging from “not at all” to “very much”. For analysis, the responses were dichotomized into two categories: “not at all” and “a little” indicating low trust, while “quite a bit” and “very much” indicating high trust. Dummy variables were created for each reference figure, with a value of 1 representing a low level of relational trust towards the respective figure.

#### Bullying and cyberbullying victimisation

The prevalence of bullying and cyberbullying victimisation was assessed by presenting respondents with a list of violent and discriminatory actions and asking if they had experienced any of these actions at their school. The list included options such as exclusion from a group, persecution through social media, sharing of personal photos or videos online without consent, threats, coercion, theft, physical assaults, sexual advances, and insults based on various factors such as ethnicity, religious beliefs, appearance, gender, sexual orientation, academic performance, and family’s economic vulnerability. A dummy variable was created during recoding, with a value of 1 assigned to individuals reporting at least one instance of victimization. Hyperconnection. This variable assessed the daily time spent on social media. Initially, the collected data were categorised into four levels of screen time: absent, attributed to individuals who do not use social media; low, encompassing those who spend between thirty and sixty minutes per day on social media; medium, corresponding to usage ranging from one to three hours per day; high, representing those who spend more than three hours daily on social media. This variable was then dichotomized to identify respondents with a high level of screen time, specifically, those who spend more than three hours per day on social media and are defined as hyperconnected. A value of 0 includes all other respondents, indicating the absence of hyperconnection.

#### Self-esteem

The Rosenberg self-esteem scale^39^ was used to assess the level of respondents’ self-esteem, defined as a positive or negative perception of oneself. This scale comprises 10 items scored on a 4-point Likert scale, ranging from “strongly disagree” to “strongly agree,” resulting in a total score between 10 and 40. Self-esteem levels were categorized as follows: scores from 10 to 15 indicated “low” self-esteem, scores from 16 to 25 reflected “healthy” self-esteem, and scores of 26 or higher represented “high” self-esteem. These classifications account for the non-linear distribution of the scale, where desirable scores generally fall within the mid-range. The internal consistency of the scale was assessed with Cronbach’s alpha, yielding a value of 0.86 in 2019 and 0.89 in 2022, indicating consistently high reliability over time.

#### Body satisfaction

Body satisfaction was measured by asking respondents whether they were satisfied with their body at the time of the interview. Responses were collected as a binary choice, indicating either satisfaction or dissatisfaction. During the recoding process, this variable was transformed into a dummy variable: a value of 0 represented dissatisfaction with their body, while a value of 1 indicated satisfaction.

#### Parental cultural status

Parental cultural status was assessed based on the educational qualifications of the respondents’ parents, following a two-stage recoding process. Initially, two separate indicators were generated to represent the educational attainment of the father and the mother, respectively. Each indicator categorized parental education into three levels: low, encompassing parents with educational qualifications up to middle school; medium, corresponding to a high school diploma; and high, representing university degrees or postgraduate qualifications. Subsequently, these individual indicators were synthesized to create a composite measure of parental cultural status comprising five levels. The low status included students whose parents both held low educational qualifications, or in the case of single-parent households, a single parent with a low educational level. Medium-low status applied to those with one parent holding a low education level and the other a medium level. Medium status encompassed cases where both parents had medium education levels or one parent had a high level and the other a low level; for single-parent households, medium status applied when the sole parent had a medium education level. Medium-high status was assigned to students with one parent having a high level of education and the other a medium level, while high status included cases where both parents had high educational qualifications or, in single-parent families, the parent had a high educational level. To facilitate analysis, the five-level classification was further simplified into three levels. The low category remained unchanged, while the medium-low and medium levels were consolidated into a single medium category. Similarly, the medium-high and high levels were merged to form the high category, resulting in a streamlined indicator of parental cultural status with three levels: low, medium, and high.

### Data analysis

The analysis of data was carried out in four successive steps employing two different software packages: SPSS (version 28, IBM, Chicago, IL, USA) and SAS (version 9.4, © 2023 SAS Institute Inc.). In the initial stage, univariate analysis was conducted to observe the frequencies of variables related to social isolation and their variation between 2019 and 2022: individuals with whom leisure time is spent, frequency of meeting friends, and concern about the absence of genuine social relationships (Table 2). Furthermore, through bivariate analysis, distributions of the inclination to spend leisure time alone, the absence of weekly in-person meetings with friends, and concern for the lack of sincere relationships were examined across specific socio-demographic variables (Table 3).

Moving to the second stage, Multiple Correspondence Analysis (MCA) was employed to explore associations among qualitative variables and identify a multidimensional space of social withdrawal phenomena. MCA, a non-parametric statistical technique developed for categorical variables similar to principal component analysis (PCA), allowed for the reduction of data dimensionality^40^. After examining the scree plot of eigenvalues, explained variance and total inertia, the primary dimensions were selected for both datasets. For the year 2019, the first two dimensions were chosen, explaining 58.3% and 40.1% of the variance, respectively, with a total inertia of 98.4%. Similarly, for 2022, the first two dimensions were selected, explaining 67.4% and 29.6% of the variance, respectively, resulting in a total inertia of 97%.

In the third stage, we applied Hierarchical Cluster Analysis (HCA) using the factorial coordinates derived from MCA for each dataset. This method, which partitions statistical units, employs Ward’s Minimum-Variance method. In Ward’s minimum-variance method, the distance between two clusters represents the ANOVA sum of squares between them, summed across all variables. At each iteration, the within-cluster sum of squares is minimized by merging two clusters from the previous generation. This approach led to the identification of three homogeneous clusters of adolescents for each year, with profiles developed based on shared characteristics: Social Butterflies, Friendship-Centric, and Lone Wolves.

Moving to the fourth stage, we conducted a descriptive analysis encompassing socio-demographic, psychological, and sociological variables to delineate the profiles of the different clusters. Lastly, to delve deeper into the Lone Wolves cluster and identify changes between 2019 and 2022, we employed the Wald test to assess the significance of the observed variations over time.

## Data Availability

Data analised in this study are available from the corresponding author upon reasonable request.
